# The Absent in Melanoma 2-Like Receptor IFN-Inducible Protein 16 as an Inflammasome Regulator in Systemic Lupus Erythematosus: The Dark Side of Sensing Microbes

**DOI:** 10.3389/fimmu.2018.01180

**Published:** 2018-05-28

**Authors:** Valeria Caneparo, Santo Landolfo, Marisa Gariglio, Marco De Andrea

**Affiliations:** ^1^Viral Pathogenesis Unit, Department of Public Health and Pediatric Sciences, Turin Medical School, Turin, Italy; ^2^Virology Unit, Interdisciplinary Research Center of Autoimmune Diseases (IRCAD), Department of Translational Medicine, Novara Medical School, Novara, Italy; ^3^Intrinsic Immunity Unit, CAAD – Center for Translational Research on Autoimmune and Allergic Disease, University of Piemonte Orientale, Novara, Italy

**Keywords:** IFN-inducible protein 16, absent in melanoma 2 (AIM2)-like receptor, inflammasome, interferon, systemic lupus erythematosus

## Abstract

Absent in melanoma 2 (AIM2)-like receptors (ALRs) are a newly characterized class of pathogen recognition receptors (PRRs) involved in cytosolic and nuclear pathogen DNA recognition. In recent years, two ALR family members, the interferon (IFN)-inducible protein 16 (IFI16) and AIM2, have been linked to the pathogenesis of various autoimmune diseases, among which systemic lupus erythematosus (SLE) has recently gained increasing attention. SLE patients are indeed often characterized by constitutively high serum IFN levels and increased expression of IFN-stimulated genes due to an abnormal response to pathogens and/or incorrect self-DNA recognition process. Consistently, we and others have shown that IFI16 is overexpressed in a wide range of autoimmune diseases where it triggers production of specific autoantibodies. In addition, evidence from mouse models supports a model whereby ALRs are required for IFN-mediated host response to both exogenous and endogenous DNA. Following interaction with cytoplasmic or nuclear nucleic acids, ALRs can form a functional inflammasome through association with the adaptor ASC [apoptosis-associated speck-like protein containing a caspase recruitment domain (CARD)] and with procaspase-1. Importantly, inflammasome-mediated upregulation of IL-1β and IL-18 production positively correlates with SLE disease severity. Therefore, targeting ALR sensors and their downstream pathways represents a promising alternative therapeutic approach for SLE and other systemic autoimmune diseases.

## Introduction

Inflammation is an innate immune response largely mediated by phagocytic cells whose goal is to protect the body from invading bacteria and viruses (1, 2). Pattern recognition receptors (PRRs) constitute a large family of molecules expressed on the cell surface and in the cytoplasm of various cell types, such as macrophages and antigen presenting cells (APC), able to interact with evolutionarily conserved pathogenic structures [i.e., pathogen-associated molecular patterns (PAMPs)], thus giving rise to multimeric protein complexes termed inflammasomes, which are then responsible for mediating a caspase-1-dependent inflammatory response (3–6). These so-called “canonical inflammasomes,” which can be triggered by a wide variety of ligands, consist of nucleotide-binding oligomerization domain (NOD)-like receptors (NLRs) and absent in melanoma 2 (AIM2)-like receptors (ALRs) (7–10). More recently, “non-canonical inflammasomes,” containing caspase-11 in mice and caspase-4/5 in humans, have also been described (11, 12).

Chronic inflammatory responses, which could last for weeks or even years, are characterized by episodes of tissue injury and healing resulting in severe tissue damage, which can eventually lead to the development of autoinflammatory/autoimmune diseases such as systemic lupus erythematosus (SLE) (13–15). This latter is an autoimmune disease characterized by a wide range of clinical and serological manifestations accompanied by a polyclonal autoimmune response against various nuclear autoantigens (16). Although genetic and environmental factors such as infections are known to be involved in the pathogenesis of SLE, the clear etiology of this disease still remains to be fully established (17).

**Figure 1:**
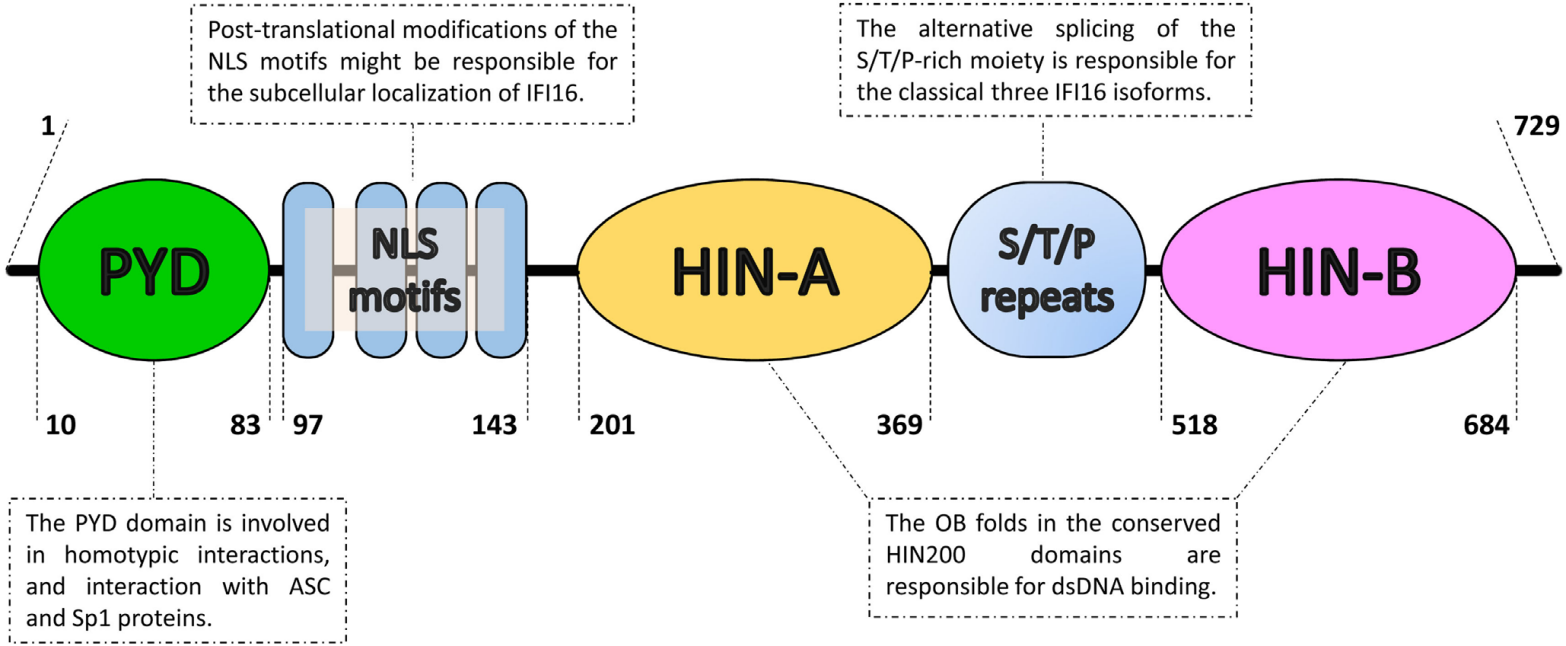
Domain organization of the IFN-inducible protein 16 (IFI16). From the N- to the C-terminal (left to right), IFI16 comprises a pyrin domain (PYD) involved in protein–protein interactions, a linker region containing four nuclear localization signal motifs (NLS) and two hematopoietic interferon-inducible nuclear protein with 200-amino-acid repeats (HIN200) domains, which is an hallmark of the absent in melanoma 2-like receptors/PYHIN proteins. The HIN200 domains include two tandem b-barrels, known as oligonucleotide–oligosaccharide-binding (OB) fold, which allow DNA docking in a non-sequence-specific manner. They are separated by serine/threonine/proline-rich (S/T/P) repeats, which are regulated by alternative mRNA splicing. The numbers represent the amino acid positions based on NCBI Reference Sequence NP_005522.2.

Despite this gap in knowledge, it is now clear that ALRs, especially the IFN-inducible protein 16 (IFI16, Figure 1), along with other inflammasome-induced inflammatory responses, contribute to the development of SLE. In this review, we will summarize recent advances on the role of the inflammasome and ALRs in SLE, which could ultimately provide the rationale for the design and development of novel therapeutic tools for the treatment of patients affected by SLE or other systemic autoimmune diseases.

## The Inflammasome

**Figure 2:**
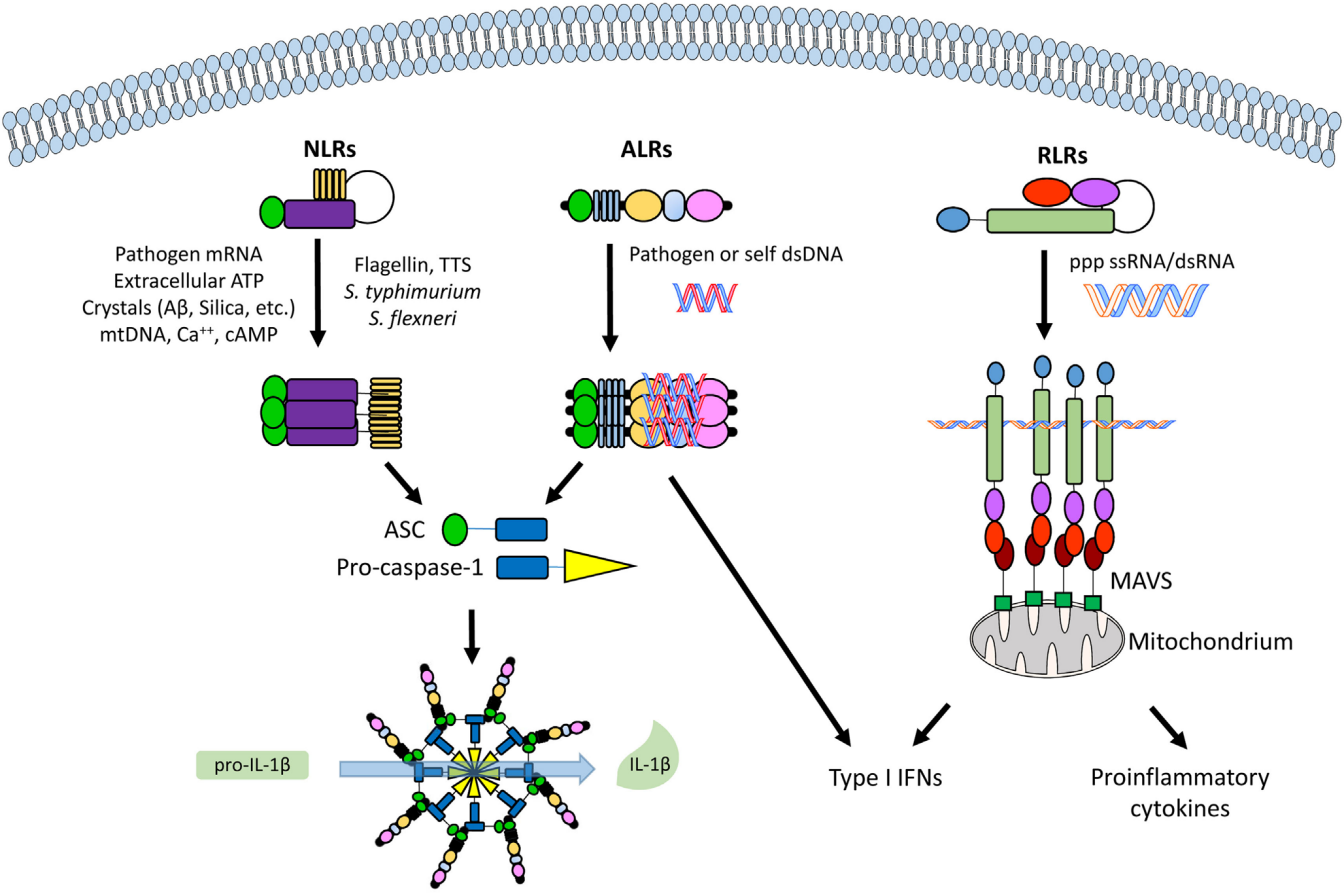
An overview of the different classes of cytoplasmic and nuclear pathogen recognition receptors (PRRs) and their involvement with inflammasome activation. From left to right, a nucleotide-binding oligomerization domain-like receptor (NLR, e.g., NLRP3), an absent in melanoma 2 (AIM2)-like receptor (ALR, e.g., IFI16), and a retinoic acid inducible gene I (RIG-I)-like receptor (RLR, e.g., RIG-I), with their commonest ligands. See text for details. Abbreviations: ASC, apoptosis-associated speck-like protein containing a caspase recruitment domain (CARD); MAVS, mitochondrial antiviral signaling protein.

The canonical inflammasome is a multimodular complex that, upon induced oligomerization, stimulates the activation of caspase-1, an enzyme primarily responsible for the release of the pro-inflammatory cytokines IL-1β and IL-18 (6). Strongly associated with the activation of the inflammasome, pyroptosis is a caspase-1-dependent type of inflammatory cell death mainly seen during intracellular infections (18). Inflammasomes specific for intracellular PAMPs involve different classes of cytoplasmic PRRs. Classically, the NLR, such as NLRP3, and the retinoic acid inducible gene I (RIG-I)-like receptor (RLR) families (Figure 2).

NLRP3 holds a C-terminal leucine-rich repeat domain, a central nucleotide-binding and oligomerization domain (NOD or NACHT), and an N-terminal pyrin domain (PYD). The NLR-associated PYD interacts with the PYD of the adaptor apoptosis-associated speck-like protein containing a caspase recruitment domain (CARD) (ASC). ASC is then able to engage caspase-1 through its CARD domain causing the oligomerization of several caspase-1 molecules that, in turn, cleave and activate each other (8). RIG-I is made of two N-terminal CARDs, a central RNA helicase domain and a C-terminal regulatory domain (CTD). As for ASC, the RIG-I CARD is a sticky domain responsible for recruiting adaptor proteins and triggering downstream pathways (19). Whereas the RNA helicase domain contains a conserved Asp–Glu–Ala-Asp motif, also known as DEAD box, and exerts ATPase activity, the CTD is responsible for binding dsRNA PAMPs (20). Following dsRNA binding and associated conformational changes, RIG-I interacts with mitochondrial outer membrane proteins called mitochondrial antiviral signaling (MAVS) through CARD–CARD interactions (21). Depending on the adaptors involved, RIG-I–MAVS interaction then results in either type I IFN (IFN-I) or pro-inflammatory cytokines production (22).

Interestingly, recent studies have shown that there also exist non-canonical inflammasomes, which, through recruitment of caspase-4/5 in human or caspase-11 in mouse, can induce caspase-1-dependent maturation and secretion of IL-1β and IL-18 (23–25). In particular, non-canonical inflammasomes appear to promote pyroptosis in a TLR4- and caspase-1-independent fashion in response to cytoplasmic Gram-negative bacteria infection (26). Although the innate immune response mediated by caspase-4/5 resembles, at least in term of outcomes, that driven by caspase-1, studies on macrophage-mediated inflammatory responses have revealed that they are indeed two quite different processes (12, 27, 28). In human cells, in fact, the CARD motif allows pro-caspase-4/5 to directly interact with lipopolysaccharide (LPS) through the lipid A moiety leading to pro-caspase-4/5 oligomerization and induction of pyroptosis coupled with secretion of IL-1β and IL-18. Adding complexity to this scenario, recent evidence has shown that murine caspase-11 activation triggers an NLRP3–ASC–caspase-1-dependent signaling pathway, also known as “non-canonical NLRP3 inflammasome activation pathway,” which is different from the aforementioned “canonical NLRP3 inflammasome activation pathway” (29). However, even though it appears that caspase-4/5 and -11 can directly detect intracellular LPS derived from Gram-negative bacteria (24, 30), the exact mechanism of the non-canonical inflammasome activation is not totally understood.

Recently, a new family of inflammasome-associated PRRs has been described, including AIM2 and IFI16, grouped as ALRs. ALRs can assemble inflammasomes that respond to DNA molecules in both the cytosol and nucleus (31–33). AIM2 and IFI16 display an N-terminal PYD and one (AIM2) or two (IFI16, Figure 1) phylogenetically conserved hematopoietic interferon (IFN)-inducible nuclear protein with a 200-amino-acid repeat (HIN200) domains at the C-terminus, thus the other name PYHIN (or PYHIN200) previously given to these proteins. Interestingly, the HIN200 domain, which consists of two oligonucleotide/oligosaccharide-binding (OB) folds (34), appears to be the major DNA recognition site (35, 36). However, due to the lack of a dedicated ATP-dependent oligomerization domain, it appears that ALRs require a longer stretch of double-stranded DNA (dsDNA) compared with that required by NLRs to bind effectively and promote oligomerization (37, 38). Notably, since DNA is a common genetic material, pathological stimulation of these nucleic acid-recognizing inflammasomes by self-DNA can lead to autoinflammatory/autoimmune diseases as well (Figure 2). In this regard, aberrant immune responses involving ALRs have long been involved in the pathogenesis of SLE, Sjogren’s syndrome (SjS), psoriasis, and systemic sclerosis (SSc) (39–45).

## Inflammasome and Autoimmunity

Although adaptive and innate responses are often opposite to each other in the immunological spectrum, they are essentially integrated in a complex system (i.e., the human body) as innate immune dysregulation (i.e., the classical driver of autoinflammatory diseases) induces autoreactive T and B cell responses (i.e., autoimmunity) (13, 46). Indeed, classical autoinflammatory diseases, such as inflammatory bowel disease (IBD), are also characterized by the presence of autoantibodies, whereas classical autoimmune conditions, such as SLE, can also display organ-specific inflammation, as in the case of lupus nephritis (LN) (47, 48).

An additional feature encompassing the full inflammatory spectrum is inflammasome activation, which is usually essential for host defense against microbes. However, recent studies have also found this activation to be responsible for or simply be associated with the pathogenesis of several diseases featuring autoinflammatory/autoimmune traits such as type 1 and type 2 diabetes, IBD, multiple sclerosis (MS), rheumatoid arthritis (RA), and SLE (49–55).

Genetic polymorphisms (SNPs) associated with autoimmune diseases have been identified in components of both NLR and ALR inflammasomes, including NLRP1, NLRP3, CARD8, IFI16, and AIM2 (52, 56–61). Several studies, most of which related to ethnicity, have highlighted an association between SNPs in inflammasome end products and autoimmune diseases such as SLE, RA, and MS (62–64). Furthermore, inflammasomes have been directly involved in autoimmunity. For example, NLRP1 is overexpressed in T and Langerhans cells in the leading edge of vitiligo skin, leading to increased IL-1β production and activation of the Th17 axis (65). Furthermore, NLRP3 expression and NLRP3-mediated IL-1β secretion are increased in RA patients (66), and NLRP3 is involved in the pathogenesis of experimental autoimmune encephalomyelitis (51). Moreover, APC with an activated NLRP3 inflammasome can trigger CD4 T cell-mediated upregulation of the chemokine receptor CCR2, which is elevated in the peripheral blood of MS patients during relapse (67). With regard to ALR family members, AIM2 is directly activated by cytoplasmic DNA (68), and a strong correlation between AIM2 overexpression and disease severity has been described in both SLE patients and mouse models (61, 69). Finally, SLE is characterized by AIM2 inflammasome-mediated production of IL-1β, triggered by accumulation of cytosolic self-DNA and IFI16-induced IFN-I release (40).

Systemic lupus erythematosus is a systemic autoimmune disease characterized by a polyclonal autoimmune response against various nuclear autoantigens (16). Although genetic and environmental factors, such as infections, have been linked to the pathogenesis of SLE, the exact etiology of this disease is still unknown (17). SLE is characterized by hyperactive autoreactive immune cells and production of many autoantibodies, immune complex (IC) formation, organ inflammation and damage. More than 200 different autoantibodies including those against single-stranded DNA and dsDNA, Ro/La antigens, and ribonuclear protein have been described in lupus patients (70, 71). Among these, the so-called antinuclear antibodies (ANAs) and anti-dsDNA antibodies, which seem to play an important role in the pathogenesis of LN, represent valuable diagnostic and prognostic markers of disease (72, 73).

Along with elevated production of autoantibodies, 50–75% of SLE adults and up to 90% of SLE children display increased IFN-I production and enhanced expression of IFN-inducible genes, which is therefore regarded as a gene expression signature of SLE (74). Notably, a few studies have shown that patients with enhanced IFN-I signature can be considered a distinct subset of SLE patients. In this context, an association between IFN signature and some clinical manifestations, such as nephritis and CNS disease, has been reported (75).

Recent studies have defined the role of autologous dsDNA in SLE pathogenesis [reviewed in Ref. (72)]. Briefly, in physiological conditions, dsDNA is localized in the nucleus and mitochondria; however, once it relocalizes into the cytoplasm and endosomes, it is rapidly degraded by DNases. In SLE patients, impaired dsDNA degradation activity coupled with defective clearance of both apoptotic cell bodies and neutrophil extracellular traps (NETs) results in self-dsDNA accumulation (76, 77). In the meantime, self-dsDNA released by apoptotic cells in the germinal center is processed by follicular dendritic cells as non-self-antigen and presented to autoreactive B cells, which as a result will survive and expand instead of being eliminated (78). Afterward, self-dsDNA together with autoantibodies triggers the formation of ICs that in turn will mediate tissue damage, stimulate pro-inflammatory cytokine production and an array of IFN-inducible genes (i.e., IFN signature). Noteworthy, self-dsDNA is mainly sensed by plasmacytoid dendritic cells (pDCs) by means of different DNA sensors, which ultimately lead to elevated IFN-I production and inflammasome activation (70).

Type I IFNs are endowed of several immune functions ranging from dendritic cell differentiation and maturation to T cells activation and induction of antibody production by B cells. IFN-I pleiotropic activities underscore the critical function of these molecules in the pathogenesis of autoimmune diseases, in particular SLE (70, 75). In parallel, inflammasome activation leads to the release of inflammatory cytokines including IL-1β and IL-18, which contributes to the maintenance of the inflammatory state followed by cell death.

However, the association between SLE and IL-1β production is highly debated. Animal models of SLE (MRL/lpr mice) have shown that IL-1β gene expression, and protein secretion is increased in the glomerular macrophages and mesangial cells of LN (79), whereas polymorphisms studies on SLE patients have led to conflicting results (80).

Altogether, these observations stress the relevant role of IFN-I alongside the other inflammatory cytokines in fine-tuning both the innate and adaptive immune responses. One can therefore easily understand how slight perturbations of the signaling pathways can lead to the dysregulation of the immune response that inevitably brings to the development of the autoimmune response.

## Role of Autologous dsDNA in SLE

The major source of autologous dsDNA, which, as mentioned earlier, plays a pivotal role in SLE pathogenesis, is represented by cells dying by necrosis, apoptosis or NETosis, with the latter being a type of cell death mediated by NETs, extrusions of intracellular material to the surrounding extracellular medium to concentrate antibacterial substances and entrap invading microorganisms (81, 82). Intriguingly, also pyroptosis, that is the type of cell death induced by the inflammasome in response to both infectious and non-infectious stimuli, has been linked to SLE initiation (83).

Apoptosis, also known as programmed cell death, is an essential mechanism of tissue homeostasis during development and aging, characterized by cell shrinkage, cytoskeleton remodeling, chromatin condensation, nuclear breakup, plasma membrane blebbing and formation of typical apoptotic bodies (84). Under normal physiologic conditions, apoptotic cells directly undergo phagocytosis by specialized cells (i.e., professional phagocytes) and are degraded within the lysosomes with no signs of inflammation or immune response. In physiological conditions, cellular membranes are well preserved and readily cleared by engulfing phagocytes (85). Unless properly cleared, the apoptotic cells undergo secondary necrosis characterized by cell membrane leakage with consequent release of intracellular contents, including autologous dsDNA (86). Notably, release of intracellular material, which ultimately contributes to the development of autoimmune diseases, can also be triggered by primary necrosis due to exogenous factors, as demonstrated both in animal models and human infections (87, 88).

NETosis, a type of cell death first associated with neutrophils, causes the extrusion of nuclear DNA, histones and granular antimicrobial proteins entrapped leading to formation of NETs (81, 89). Yet, mounting evidence has shown that other cell types, including eosinophils and mast cells, can undergo cell death through a similar mechanism. Therefore, NETosis appears not be limited to neutrophils and should therefore be regarded as a new type of cell death that generally causes the release of extracellular traps (90). Physiologically, monocyte-derived phagocytes clear NETs efficiently thanks to C1q- and DNase I-mediated extracellular preprocessing of NETs. After ingestion by phagocytes, NETs are degraded in the lysosomes. Remarkably, this entire process is immunologically silent since the uptake of NETs by macrophages does not seem to stimulate pro-inflammatory cytokine secretion (91). On the other hand, impaired clearance of NETs by phagocytes can lead to the accumulation of several autoantigens including self-dsDNA (92), thereby increasing the chance of anti-dsDNA antibody formation, although a study on an animal model of SLE showed a protective role of NETs (93).

A particular type of NETosis, mitochondrial NETosis, causes the release of mitochondrial DNA (mtDNA) from neutrophils following the mitochondrial production of ROS. Since mitochondria share several features with bacteria, including a circular genome carrying unmethylated CpG dinucleotide repeats, mtDNA is similarly immunogenic and may promote inflammation through surface and endoplasmic TLR9 binding. Moreover, IL-1β production can also be driven by cytosolic release of mtDNA, dominantly acting on NLRP3/AIM2 inflammasomes (94). Interestingly, NETs from low-density granulocyte of SLE patients are highly enriched in mtDNA compared with NETs from healthy controls neutrophils (95), whereas abnormal extrusion of oxidized mtDNA from SLE patient neutrophils may triggers a pathogenic interferogenic response (96). Finally, mtDNA and autoantibodies against it are present in elevate levels in SLE and in particular in LN, where levels correlate with activity index better than anti-dsDNA (97).

Altogether, these findings indicate that cell death-originating self-dsDNA plays a crucial role in SLE pathogenesis.

## Environmental Factors Triggering IFN-I Production and Inflammasome Activation in SLE

We have beforehand described that DNA from dying cells, as well as DNA from microbial pathogens, is strong immune stimulants that can accumulate in the cytosol and activate the production of various immune system modulators, including IFN-I. This pathway is critically dependent on a protein known as stimulator of interferon genes (STING) (98), which indirectly responds to DNA through the cyclic dinucleotide 2′,3′-cGAMP, produced upon the stimulation of the enzyme cyclic GMP-AMP synthase (cGAS) (99). In turn, the 2′,3′-cGAMP-related activation of STING induces a conformational change which is thought to mediate the phosphorylation and activation of interferon regulatory factor 3 (IRF3), a transcription factor for various gene targets, including but not limited to IFN-I (100).

It is becoming increasingly clear how several environmental factors that can promote IFN-I production are also able to induce an SLE syndrome as well as cause a flare of this disease. One of these agents is represented by ultraviolet B (UVB) light, which has been shown to trigger SLE flares and induce severe systemic manifestations including cutaneous reactions (101). Interestingly, all UVB light-induced exacerbations are associated with enhanced levels of IFN-I and -III along with pro-inflammatory cytokines (102, 103). In this regard, UVB light can promote redistribution of nuclear antigens on the cell surface and keratinocyte apoptosis (104). Furthermore, additional inflammatory cells, recruited by type III IFN into the skin, are likely responsible for priming activated pDCs to produce higher levels of IFN-I. Consistently, UV irradiation of keratinocytes has been shown to activate the STING/IRF regulatory axis in response to cytosolic DNA due to the loss of the STING negative regulator Unc51-like kinase 1 (105).

Systemic lupus erythematosus onset along with disease flare is also frequently associated with infections. Although many viruses and bacteria have been implicated in SLE pathogenesis (88, 106, 107), no specific etiologic pathogen has thus far been identified. Inflammation, as part of the innate immune response, is triggered when PAMPs are recognized by PRRs, which can be either associated with the cell membrane or located within the cell in the cytosol or nucleus. There is a growing number of identified PRRs, including toll-like receptors (TLRs) and various intracellular nucleic acid receptors. The signaling pathway leading to IFN-I production or inflammasome activation strictly relies on the PRR repertoire of the responding cell type and the subcellular localization of the immunostimulatory nucleic acid. TLR3, TLR7/8, and TLR9, present in immune cells (i.e., pDCs and monocytes), sense dsRNA, ssRNA, and DNA containing CpG motifs (108, 109). Another group of PRRs (i.e., the RLRs) include the cytosolic RNA receptor RIG-I and the melanoma differentiation factor 5, and are responsible of detecting dsRNA and ssRNA molecules in the cytoplasm of cells infected with RNA viruses (20, 110–112). In addition, several DNA sensors located in both the cytosol and the nucleus have been described. These include cGAS (113), DNA-dependent activator of IFN-regulatory factors (DAI) (114), AIM2 (115), IFI16 (116, 117), NLRs (118), and DEAD/H-box helicase 41 (DDX41) (119). Binding of these DNA sensors to their ligands activates signaling pathways, including TLR9-, STING-, and inflammasome-dependent pathways, which not only induce production of IFN-I but also promote inflammatory gene expression and inflammasome-associated cell death (i.e., pyroptosis). In physiological conditions, these intracellular sensors and related pathways are tightly regulated to impede the development of autoimmunity (120), which would otherwise take place due to uncontrolled recognition of self-nucleic acids (121, 122).

Upon PAMP recognition, the intracellular receptors assemble cytoplasmic platforms known as myddosomes and inflammasomes, which are supramolecular organizing centers regulating the inflammatory and immunoregulatory response following microbial detection. Specifically, TLRs initiate a toll/interleukin-1 receptor domain-containing adapter protein (TIRAP)-dependent assembly of the myddosome, which consists of the adaptor MYD88 and several serine/threonine kinases of the IL-1 receptor-associated kinase family (123). As stated previously, the canonical inflammasome contains a DNA sensor protein, the adaptor protein ASC and procaspase-1. Upon inflammasome assembling, activation of caspase-1 converts the immature IL-1β and IL-18 into mature secreted forms (124). Importantly, different NLR family members, such as NLRC4, NLRP1, and NLRP3 and the two ALR family members AIM2 and IFI16 have been shown to be differentially stimulated in a ligand-specific fashion.

Recently, it has been demonstrated that the canonical inflammasome pathway can be by-passed by the non-canonical one, which as stated previously consists of a complex formed by procaspase-11 and bacterial LPS activated in mouse macrophages. Consistently, caspase-4 and caspase-5, the human counterpart of mouse caspase-11, can interact directly with intracellular LPS and activate the non-canonical inflammasome in human myeloid cells (12, 23).

Two important features distinguish myddosomes from inflammasomes: (1) inflammasomes do not trigger gene activation at the transcriptional level, but rather induce inflammation by promoting the release of preexisting immature cytokines; (2) inflammasomes activating PRRs are localized in the host cytosol, which is rarely attacked by non-pathogenic bacteria. Therefore, inflammasomes are generally assembled when intracellular PRRs interact with pathogenic bacteria in the cytosol. By contrast, TLRs, which are localized on the cell surface, cannot distinguish whether PAMPs originated from pathogenic or non-pathogenic microorganisms.

Thus, taken together, these findings suggest a scenario where the redundancy of PAMPs sensing immune receptors may easily lead to dysregulation of the immune response when not regulated properly.

## AIM2-Like Receptors: Inflammasome Activators and IFN-I Production Regulators

**Figure 3:**
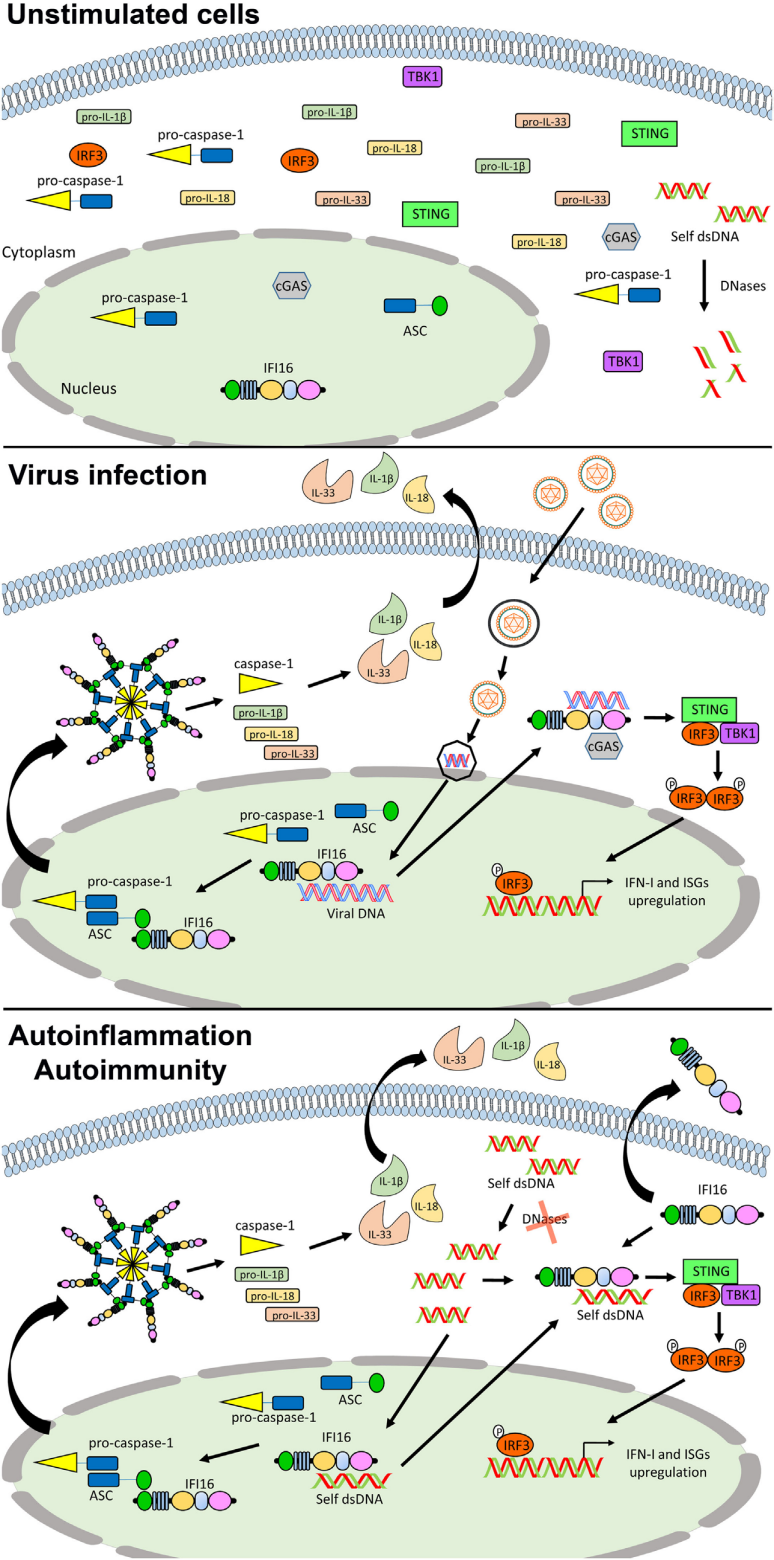
Role of IFN-inducible protein 16 (IFI16) as inflammasome regulator in viral infections and autoimmunity. In unstimulated cells, IFI16 is mainly nuclear (upper panel). Following viral DNA recognition and binding, IFI16 can induce the activation of the canonical inflammasome through the recruitment of ASC and pro-caspase 1, and the production of type I IFN (IFN-I) through the STING–IRF axis (middle panel). In the course of autoimmune (e.g., systemic lupus erythematosus) and autoinflammatory conditions, following the recognition of self-DNA, IFI16 might be responsible for the production of pro-inflammatory cytokines and IFN-I through the same pathways. Moreover, its aberrant expression can also lead to the extracellular leakage causing amplification of the inflammatory signals and production of protective autoantibodies (lower panel). See text for details. Abbreviations: cGAS, cyclic GMP-AMP synthase; IRF3, interferon regulatory factor 3; ISG, interferon stimulated genes; STING, stimulator of interferon genes; TBK1, TANK-binding kinase.

The PYHIN (or PYHIN200) family encodes evolutionary related human (i.e., IFI16, IFIX, MNDA, and AIM2) and murine (i.e., Ifi202a, Ifi202b, Ifi203, Ifi204, Ifi205/D3, and Ifi206) proteins (116–118). Increasing evidence has shown that these proteins may act as regulators of a wide range of cell functions, such as differentiation, proliferation, senescence, apoptosis, and inflammasome assembly (117, 125–130). Recently, two members of the human family, IFI16 and AIM2, have been implicated in the recognition of pathogen DNA and classified into the ALR group, still maintaining their peculiarity. In normal conditions, expression of the nuclear phosphoprotein IFI16 is limited to vascular endothelial cells, keratinocytes, and hematopoietic cells (131). Following activation by pathogen DNA, IFI16 translocates into the cytoplasm, triggers type I IFN production, cytokines, and eventually cell death (Figure 3). By contrast, AIM2, upon binding DNA in the cytosol, stimulates inflammasome activation in the absence of type I IFN production.

From a structural point of view, IFI16 harbors an N-terminal PYD and two C-terminal HIN200 domains (see Figure 1 for details). While AIM2 uses its PYD to interact with the inflammasome component ASC, which also contains a PYD (31, 33, 68), the direct interaction between IFI16 and ASC is still matter of debate. Nevertheless, IFI16 has been reported to induce ASC-dependent inflammasome activation during infection with some nuclear DNA viruses (32, 132, 133). Following viral DNA recognition in the nucleus, the IFI16-ASC-procaspase-1 inflammasome formation is induced. The complex is then released in the cytoplasm, where processing of pro-IL-1β into active IL-1β occurs.

Moreover, IFI16 is also an inducer of IFN-β in response to intracellular DNA. RNA interference-mediated depletion of IFI16 or its presumed mouse ortholog p204 has revealed that both proteins are required for a functional IFN response to transfected dsDNA or infection with HSV-1 in various cell types, including human and mouse monocytic cell lines (134), mouse corneal epithelial cells (135), human primary and immortalized fibroblasts (136, 137), human primary macrophages (138), neutrophils (139), and dendritic cells (140). In this regard, IFI16 has been shown to interact with STING, leading to phosphorylation and nuclear translocation of IRF3 *via* the IFI16–STING–TBK signaling axis, resulting in IFN-β production during HSV-1 infection (137). Moreover, IRF3 activation has been also demonstrated following direct cooperation between IFI16 and cGAS, by a mechanism in which cGAS promotes IFI16 stability in response to incoming nuclear HSV DNA, rather than through the production of 2′,3′-cGAMP (141) (Figure 3).

As aforementioned, IFI16 is unique among DNA sensors as it shuttles between the nucleus and the cytoplasm and is predominantly nuclear at steady state. Furthermore, IFI16 is able to sense DNA in both compartments depending on the localization of its DNA ligands (134, 137, 138). Thus, the ability of IFI16 to detect DNA viruses, such as HSV-1 in the nucleus, appears to contradict the long-held assumption that foreign DNA is sensed merely because of its cytosolic localization. Interestingly, the conserved HIN200 domains of the IFI16 protein are responsible for the interaction with oligonucleotide/oligosaccharide moieties (142). To what extent IFI16/p204 is involved in the sensing of DNA during infection with viruses or intracellular bacteria *in vivo*, and what domains are indispensable for recognition, awaits the generation of mice lacking this receptor. However, the structural studies elucidating the DNA binding of IFI16 have improved our understanding on non-self-DNA sensing and IFI16 localization. Few more issues concerning the nuclear/cytosolic interaction of STING and IFI16 and activation of inflammasome remain unanswered, mainly related to the different cellular and infection models analyzed so far.

IFN-inducible protein 16 has also been described to play a role in the DNA damage response (143, 144) and promote apoptosis and senescence (145–148). Recent reports have implicated IFI16 in autoimmunity, pointing to a role of PYHIN proteins in the pathogenesis of human autoimmune disease. Since the IFN system is largely regarded as playing a key role in autoimmune disorders including SLE, SSc, and SjS (75, 149, 150), it is possible to hypothesize that also PYHIN may play a causative role in autoimmunity thanks to its ability to induce apoptosis and trigger an inflammatory response (Figure 3). It follows that during systemic autoimmune conditions of tissue injury and apoptosis the exposure of autoantigens leads inevitably to breaking of tolerance and dysregulation of the immune response. Under physiological conditions, dead cells and tissue debris are normally cleared by the monocyte/macrophage system. However, under conditions that hamper clearance of apoptotic bodies by phagocytes, chronic exposure of autoantigens, including PYHIN proteins, may lead to the development of autoimmunity. Consistent with this scenario, various autoantigens and corresponding autoantibodies have been identified in the sera of patients affected by different systemic autoimmune diseases, such as SLE, SjS, and SSc.

## Novel Functions for IFI16 to Trigger Inflammation

We have previously demonstrated that IFI16 is a key component for the tight regulation of cellular and viral promoters, through physical interaction with the nuclear transcription factor Sp1 and regulation of NF-κB pathway (151, 152). As inducer of pro-inflammatory molecules (e.g., ICAM-1, RANTES, and CCL20) and apoptosis in primary endothelial cells, IFI16 might be active during the initial phases of the inflammatory processes paving the way to the onset of autoimmunity (145). In addition, IFI16 has been shown to translocate in the cytoplasm of normal keratinocytes following UVB-induced cell injury and be subsequently released in the extracellular milieu (104). *In vivo*, the expression of IFI16 is significantly increased in all layers of the epidermis from patients affected by SLE or SSc, whereas in the epidermis from healthy control subjects IFI16 expression is only found in the basal layer. In the same setting, the dermal inflammatory infiltrate has been found positive for IFI16 staining indicating that IFI16 is aberrantly expressed also in lymphocytes, fibroblasts, and endothelial cells. Similarly, we and others have also recently demonstrated that IFI16 is aberrantly expressed in the intestinal mucosa of patients affected by IBD, where dysregulation of host–microbial interactions has been shown to play a major pathogenic role (153, 154). In addition, we and others have evaluated the etiopathogenic role of PYHIN proteins in the development of SLE in human pathology as well as in mouse models (Table 1). In this regard, we have found that IFI16 overexpression in primary human umbilical vein endothelial cells (HUVECs) efficiently inhibits tube morphogenesis *in vitro*, triggers production of pro-inflammatory molecules and leads to cell death by apoptosis, suggesting that IFI16 might induce inflammation along with other detrimental cellular pathways primarily involved in autoimmunity (145, 155).

**Table 1:** Summary of IFN-inducible protein 16 (IFI16) correlations with systemic lupus erythematosus (SLE) and other autoimmune diseases.

Disease	Observation	Reference
Systemic lupus erythematosus	First description of anti-IFI16 antibodies in the sera SLE patients	(159)
	Presence of anti-IFI16 antibodies detected by SEREX in the sera of SLE patients	(43)
	Increased expression of IFI16 in the skin of SLE patients and detection of anti-IFI16 antibodies by ELISA	(41)
	Increased IFI16 mRNA levels in leukocytes from SLE patients	(55)
	IFI16 overexpression and redistribution in the skin of SLE patients	(104)
	High significant levels of circulating IFI16 protein in the sera of SLE patients	(155)
	High serum titers of anti-IFI16 antibodies inversely correlated with proteinuria and C3 hypocomplementemia	(158)

Sjögren’s syndrome	Presence of anti-IFI16 antibodies detected by SEREX in the sera of Sjogren’s syndrome (SjS) patients	(43)
	Significant levels of circulating IFI16 protein in the sera of SjS patients	(155)
	*De novo* expression of IFI16 in ductal and acinar epithelial cells in salivary glands	(39)
	High serum titers of IFI16 antibodies against an epitope outside the N-terminus of the protein	(160)

Systemic sclerosis	Presence of anti-IFI16 antibodies detected by SEREX in the sera of systemic sclerosis (SSc) patients	(43)
	Increased expression of IFI16 in the skin of SSc patients and detection of anti-IFI16 antibodies by ELISA	(41)
	Anti-IFI16 antibodies associated with the limited cutaneous form of the disease in patients negative for the classical serological markers	(161)
	Significant levels of circulating IFI16 protein in the sera of SSc patients	(155)

Rheumatoid arthritis (RA)	Presence of anti-IFI16 antibodies detected by SEREX in the sera of RA patients	(43)
	High levels of circulating IFI16 protein in the sera of RA patients	(155)
	Increased levels of both anti-IFI16 antibodies and circulating IFI16 in the sera of RA patients, IFI16 protein correlating with RA-related pulmonary disease	(157)

Inflammatory bowel disease	*De novo* overexpression of IFI16 in colonic epithelial cells of inflammatory bowel disease (IBD) patients	(153, 154)
	Detection of anti-IFI16 antibodies by ELISA in the sera of IBD patients	(153)

Psoriasis	IFI16 upregulation in psoriatic skin lesions, with cytoplasmic localization	(44)
	IFI16 upregulation in keratinocytes is induced by psoriasis-related cytokines, including IFN-γ, TNF-α, IL-17, and IL-22	(45)

In another context, IFI16 has been shown to restrict human cytomegalovirus (HCMV) and papillomavirus replication through different mechanisms (152, 156). Interestingly, IFI16 has been observed entrapped in HCMV virions undergoing cell egression (116). Consistent with our results, Singh et al. have demonstrated that IFI16 is aberrantly expressed in the cytoplasm of KSHV latently infected cells, wrapped up in exosomes and then released extracellularly (133). However, since IFI16 was originally identified as a molecule localized in intracellular compartments, in particular the nucleus, all studies on IFI16 were subsequently limited to determine the biological and physiological activity of this protein exclusively within the cellular compartment, thus disregarding a possible role of extracellular IFI16 as pro-inflammatory trigger. To fill this gap, we sought to determine the effects of extracellular IFI16 protein on HUVECs. Surprisingly, we observed a cytokine-stimulating activity of extracellular IFI16 (rIFI16) on primary endothelial cells, which led to the production and secretion of pro-inflammatory cytokines such as IL-6, IL-8, CCL2, CCL5, and CCL20. Moreover, we found that rIFI16 protein, alone or in synergy with LPS, acted by propagating “danger signals” through a MyD88-dependent TLR pathway (126).

Altogether, these results unveil a novel function of extracellular IFI16 at the endothelial interface, which might explain the ability of this protein to induce endothelial cell activation and injury during systemic inflammation.

In summary, IFI16 can promote inflammation by (1) acting as regulator of transcription factors to activate expression of genes encoding pro-inflammatory cytokines; (2) activating type I IFN production following translocation into the cytoplasm; and (3) binding to cells such as endothelial cells and keratinocytes, once released in the extracellular milieu, to activate production of pro-inflammatory chemokines and cytokines. Concomitantly, IFI16 leakage into the extracellular milieu leads to tolerance breaking and autoantibody production.

## Anti-IFI16 Antibodies and Their Relation to SLE Characteristics

We and others have previously reported the presence of anti-IFI16 antibodies in sera of patients suffering from various autoimmune diseases such as SLE, SjS, AR, SSc, and IBD (39, 41, 153, 157–161) (Table 1). Among these latter, SLE stands out as the disease where IFI16 autoantibodies have been more thoroughly characterized. This aspect is of paramount importance in view of the prognostic and diagnostic relevance of other SLE autoantibodies such as ANAs and autoantibodies against Ro/SSA and La/SSB ribonucleoproteins (162). However, not all autoantibodies seem to play a causative role in autoimmunity as autoantibodies against chromatin molecules, such as HMGB1, exert a protective effect in animal models of autoimmune disease (163). Thus, new criteria for autoantibodies classification based on both their functionality and ability to trigger or dampen immunologic disturbances are clearly needed.

With regard to IFI16, it is conceivable to hypothesize that the previously described over- or aberrant expression and mislocalization of this nuclear protein, earlier in the cytoplasm and later on in the extracellular milieu, might lead to loss of tolerance and development of anti-IFI16 antibodies, as demonstrated in skin lesions from SLE patients and in keratinocytes cultured *in vitro* under conditions of UVB light-induced cell injury (104).

Although the occurrence of anti-IFI16 antibodies in SLE patients has long been known, their associations with clinical and serological parameters of SLE are still under debate. To address this aspect, we have recently set out to determine the prevalence of anti-IFI16 autoantibodies in a population of SLE patients from northern Italy (158). Specifically, in a cross-sectional study, we compared anti-IFI16 antibody levels of SLE patients at various stages of disease with those of patients with non-SLE primary glomerulonephritis (GN) or healthy individuals. Remarkably, we measured significantly higher anti-IFI16 titers in SLE patients compared with both disease and control groups, and, according to cutoff levels, we were able to estimate that more than 60% of the SLE patients tested positive for anti-IFI16 autoantibodies compared with just 24% of patients with primary non-SLE GN and 5% of healthy individuals. Of note, in this SLE cohort, univariate analysis showed that autoantibodies to IFI16 were inversely associated with proteinuria, whereas multivariate analysis confirmed a reduced risk of proteinuria for anti-IFI16-positive patients despite renal function. Furthermore, an inverse association between anti-IFI16 and C3 hypocomplementemia was also observed. In this regard, the association of anti-IFI16 antibodies with reduced C3 hypocomplementemia was independent of the disease activity parameters SLEDAI and anti-dsDNA. The described inverse associations between anti-IFI16 positivity, proteinuria, and C3 hypocomplementemia, together with the observation that nephritis does not occur in other systemic autoimmune diseases characterized by high titers of anti-IFI16 antibodies such as SjS and SSc, imply that ultimately these antibodies do not play a relevant role in the pathogenesis of renal inflammation in SLE, but rather most likely prevent complement consumption. Thus, based on these findings, it is likely that the occurrence of IFI16 autoantibodies might protect from the detrimental activity of the free circulating IFI16 protein, exerting beneficial functional effects rather than pathogenic ones.

Consistent with the data obtained in SLE patients, in previous studies, we found a significant prevalence of anti-IFI16 antibodies in SSc, which was more evident in the more benign limited cutaneous form of this disease (42). More recently, we have shown that enhanced titers of anti-IFI16 in IBD patients undergoing infliximab therapy correlates with a more favorable outcome of the disease (153), which can be partly explained by the protective role exerted by these antibodies against the progression of the autoimmune process.

## Conclusion and Perspectives

In the last decade, we have greatly expanded our knowledge of the relationship between aberrant innate immune response and development of autoinflammatory/autoimmune diseases such as SLE. Specifically, we now know that multiple inflammasome-induced inflammatory responses correlate with the development of SLE. In this regard, the ALR family member IFI16 has been found aberrantly expressed in various target tissues of a range of autoimmune diseases, including SLE skin, SjS salivary glands, and IBD colonic epithelium. With this scenario in mind, the occurrence of anti-IFI16 antibodies is likely due to the response of the immune system to IFI16 protein release through one of the aforementioned cell death mechanisms. Alternatively, the presence of anti-IFI16 autoantibodies could be the result of IFI16 translocation from the nucleus to the cytoplasm and, eventually, being secreted into the extracellular milieu where it is recognized by the immune system. In addition, the observation that IFI16 enhances the inflammation response against microbial patterns, such as bacterial LPS, is highly suggestive of a role of ALRs also in non-canonical inflammasome-mediated signaling.

Overall, understanding the role of ALRs in SLE pathogenesis and chronic inflammation would contribute to the development of novel therapeutic options, which may not only be limited to the treatment of patients affected by systemic autoimmune disease but also to cure conditions in which prolonged inflammatory flares progressively lead to organ-specific disorders (e.g., cancer).

## Author Contributions

VC, SL, MG, and MDA have made a substantial, direct, and intellectual contribution to the work and approved it for publication.

## Conflict of Interest Statement

The authors declare that the research was conducted in the absence of any commercial or financial relationships that could be construed as a potential conflict of interest.
